# Near-infrared fluorescence guided surgery: State of the evidence from a health technology assessment perspective

**DOI:** 10.3389/fsurg.2022.919739

**Published:** 2022-07-26

**Authors:** Tibor Géczi, Zsolt Simonka, Judit Lantos, Melinda Wetzel, Zsolt Szabó, György Lázár, József Furák

**Affiliations:** ^1^ Department of Surgery, Faculty of Medicine, University of Szeged, Szeged, Hungary; ^2^ Department of Neurology, Bács-Kiskun County Hospital, Kecskemét, Hungary; ^3^ Department of Anesthesiology, Faculty of Medicine, University of Szeged, Szeged, Hungary; ^4^ Institute of Surgical Research, Faculty of Medicine, University of Szeged, Szeged, Hungary

**Keywords:** lung surgery, VATS, near-infrared fluorescence-guided surgery, indocyanine green, segmentectomy, sentinel lymph node

## Abstract

Different applications of near-infrared fluorescence-guided surgery are very promising, and techniques that help surgeons in intraoperative guidance have been developed, thereby bridging the gap between preoperative imaging and intraoperative visualization and palpation. Thus, these techniques are advantageous in terms of being faster, safer, less invasive, and cheaper. There are a few fluorescent dyes available, but the most commonly used dye is indocyanine green. It can be used in its natural form, but different nanocapsulated and targeted modifications are possible, making this dye more stable and specific. A new active tumor-targeting strategy is the conjugation of indocyanine green nanoparticles with antibodies, making this dye targeted and highly selective to various tumor proteins. In this mini-review, we discuss the application of near-infrared fluorescence-guided techniques in thoracic surgery. During lung surgery, it can help find small, non-palpable, or additional tumor nodules, it is also useful for finding the sentinel lymph node and identifying the proper intersegmental plane for segmentectomies. Furthermore, it can help visualize the thoracic duct, smaller bullae of the lung, phrenic nerve, or pleural nodules. We summarize current applications and provide a framework for future applications and development.

## Introduction

Surgery is the frontline treatment for most types of solid tumors (1). Obtaining negative margins of excision is essential to improve the patients' survival rate and quality of life. Complete removal of tumor tissue is critical for prolonged survival (2–4). Despite various advancements in preoperative imaging, the rate of positive resection margins has not decreased in recent years (4). While preoperative imaging modalities, such as computed tomography (CT), magnetic resonance imaging (MRI), and positron emission tomography-CT, have shown significant development within the past decades, the intraoperative evaluation of the resection margins is still based on surgeon's inspection and finger palpation. Radiography or ultrasound imaging can be used as additional tools for intraoperative guidance, but these tools are often insufficient and highly operator-dependent (5), mainly in thoracic surgery. Intraoperative MRI and CT can also play a significant role; however, these systems are very complex and expensive and are used mainly for neurosurgery at major, selected, leading centers (6).

During the last 2 decades, the intraoperative use of invisible near-infrared (NIR) fluorescence imaging has started to find its role in the surgical theater, filling the gap between preoperative imaging and intraoperative findings (7). NIR fluorescence imaging systems use a special camera to detect the infrared light emitted by a fluorescent dye after excitation by a specified infrared light. All these systems can be integrated into a camera used during open surgery or within laparoscopic or robotic instruments. Nowadays, different NIR systems have been developed, such as „Novadaq SPYTM system, Hamamatsu's Photodynamic Eye, ArtemisTM, Fluoptics' Fluobeam, functional intraoperative FMI systems: FLARETM imaging system” etc (7).

There are several advantages to using NIR light-imaging systems. While visible light can travel to the tissue only a few microns, NIR light (700–900 nm) can penetrate even up to centimeters through different tissues (8). As the tissue shows minimal autofluorescence in the infared spectrum, the so-called signal-to-background ratio can be maximized, when using NIR fluorescent dyes, creating “white stars in a black sky” (9), achieving optimal contrast during imaging. In addition, using this technique there is no use of ionizing radiation, making it basically safe. Furthermore, as NIR light is not visible to the human eye, it does not affect the surgeon's vision (7).

## Fluorophores used for NIR fluorescence guided surgery

### Indocyanine green (ICG)

Indocyanine green (ICG) is the dye that is used most frequently for NIR guidance (Supplementary Table S1). It is a water-soluble, amphiphilic, tricarbocyanine fluorophore with a molecular weight of approximately 775 D and an absorption and fluorescence spectrum in the NIR region (10). When administered systemically, the ICG forms nanoparticles by rapidly binding to plasma proteins and lipoproteins. The liver takes up and excretes more than 80% of the available ICG into the bile within 18 h of administration (10). ICG is safe at systemic doses as high as 5 mg/kg, although some cases of anaphylaxis have been reported (11). Wavelengths of excitation and emission are approximately 805 and 830 nm, when ICG is dissolved in blood (11). The 830 nm wavelenght of emission spectrum of ICG shows tissue penetration to up to 15 mm, and there is almost no autofluorescence from endogenous tissues (12). Having of the amphiphilic features and protein-binding attributes of ICG, it is able to migrate within lymphatic veins. Furthermore, ICG is quite cheap, non-toxic, Food and Drug Adminsitration (FDA)-approved, and readily available, making it an optimal dye for NIR fluorescence guidance. Currently, the FDA specifically approves of using ICG for cardiac output tests, hepatic function tests, and ophthalmic angiography (11).

However, ICG also has some disadvantages, such as moderate photostability, a relatively narrow fluorescence quantum yield, a high propensity to bind plasma proteins and aggregation in water solutions (13). Most of all, ICG is not able to bind specifically to tumor cells and accumulates only aspecifically in tumor tissue (14).

However, there is a way to utilize the advantageous features of ICG and offset its disadvantages, that is, to design NIR nanocomplexes created with ICG, highly selective for tumors, showing high tumor-to-background ratios, and negligible toxicity (15–19). The targeting of ICG with nanocomplexes provides its protection and extends its circulation time, while the connection of the appropriate target proteins leads to tissue-specific labeling. Using nanoparticles, objects between 5 and 200 nm in size have increased efficacy, specificity, and biostability, especially in terms of the fluorescent agent. The second generation so-called stealthy nanoparticles underwent surface changes allowing them to avoid the immune cells; therefore, the plasma half-life is significantly increased (20). Third-generation nanocomplexes are stealthy and targeted, and their surfaces are functionalized with biologically active proteins that recognize specific tumor molecules (20).

### Passive tumor targeting

Passive targeting of tumor tissue by nanocomplexes is based on the enhanced permeability and retention (EPR) effect. The EPR effect is derived from incomplete and pokey vessel formation in solid tumors with relatively hugh gaps between endothelial cells, resulting in accumulation of nanomolecules (21–23). Tumor-induced neovascularization is poorly structured, leading to increased extravasation of molecules that can go through the discontinuities of the endothelial layer, ranging from 200 to 2,000 nm. After extravasation, larger particles are preferentially retained in the tumor, thanks to the absence of functioning lymphatic system (24). However, due to the variability of the vascular system, tumor stroma, and lymphatic drainage, the EPR effect depends on the tumor type and location. Thus nanoparticles are useful methods of tumor targeting, but can be used only in certain cases. For example, EPR is more pronounced in small tumors, which is probably due to the higher density of vessels than that in large tumors with frequently necrotic areas (25).

There are two main types of ICG nanoparticles: inorganic nanoparticles and mesoporous silica nanoparticles, which have excellent biocompatibility and easy functionalization with different compounds (26). Calcium phosphate nanocomplexes can be applied during *in vivo* tumor imaging and drug, gene, or small interference RNA delivery (27). In addition, these nanoparticles are non-toxic (27). Furthermore, a magnetic carbon nanoparticle with ICG was developed for combined fluorescence and MRI imaging (28). Some organic nanoparticles have been developed over the years, such as poly (lactic-co-glycolic acid) (PLGA) carriers, liposomes, and nanoparticles, in which ICG was encoated in the centre of a polymeric micelle, self-assembled from amphiphilic polyethylene glycol (PEG)-polypeptide hybrid triblock copolymers of poly(ethylene glycol)-b-poly(L-lysine)-b-poly (L-leucine) (PEG-PLL-PLLeu), with PLLeu as the hydrophobic core and PEG as the hydrophilic shell, which has been effective in mice for targeting non-small cell lung carcinoma (29). Studies have demonstrated that encapsulation of ICG into polymer-based nanocomplexes positively affects the nature of ICG (30–32). Organic nanocomplexes show great passive tumor-targeting feature and extended circulation time. They are released gradually and slowly, which does not allow the strong binding of ICG to nonspecific proteins and the rapid elimination *via* the kidneys ((30–32)

### Active tumor targeting

The use of nanocomplexes loaded with ICG for active tumor targeting is based on two elements: the target molecule and recognition of the target. The target protein must be present on the cell surface, be characteristic to the tumor, or leastwise be expressed more pronounced in tumors (33, 34). Numerous tumors, mainly breast and brain tumors, overexpress folate receptors, which makes folic acid an adequate ligand active targeting of these tumors (33, 34). ICG nanocomplexes with hyaluronic acid allow passive targeting by the EPR effect due to nanoparticles. And these nanocomplexes make possible active targeting as well, thanks to the affinity of hyaluronic acid for CD44 (33, 34).

The new active tumor-targeting strategy involves the conjugation of ICG nanoparticles with antibodies. Antibodies against human epidermal growth factor receptor 2 (HER-2) are in the center of interest for the imaging of active tumor targeting (35, 36). For example, HER-2 plays role in the developement of different tumors such as non-small cell lung cancer. Different forms of nanocomplexes have been examined, such as nanocapsules, erythrocytederived transducers (37), and silica nanocomplexes (36). All these forms have showed significantly higher fluorescence in cells overexpressing HER-2 than in tumor cells underexpressing HER-2 (36). ICG can be conjugated with other antibodies such as daclizumab, trastuzumab, and anti-integrin αvβ6 antibodies. Furthermore, ICG can be conjugated with antibody fragments with desirable pharmacokinetic characteristics. Because of this, Sano et al. published an activatable optical imaging molecule made of a PSMA-targeted cys diabody joined with ICG (38). This probe is activated solely when connects to the tumor, which results significant signal-to-background ratios. Finally, ICG can be conjugated with other ligands, such as chlorotoxin (39), which is a scorpion venom derivate and has binding proteins in several solid tumors (40).

### 5-Aminolevulinic ACID

5-Aminolevulinic acid (5-ALA) is the main substrate for protoporphyrin synthesis and has been used during fluorescence imaging for tumor detection and during photodynamic therapy for several years. 5-ALA is transformed into heme by the ferrochelatase enzyme. The expression and density of this enzyme is low in tumor cells, leading to the accumulation of Pp-IX. The differences in concentration and pharmacokinetics between normal and tumor cells unburden the use of 5-ALA for diagnosis and treatment of malignant tumors (41).

### EC17

EC17 is one of the folate analoges joined with fluorescein isothiocyanate with absorption and fluorescence spectrum in the near-infrared and visible-light (42). EC17 is a good choice because the folate receptor-alpha (FRα) is highly expressed in some epithelial carcinomas, including pulmonary adenocarcinomas (43, 44). The main disadvantage of EC17 in preclinical studies are its poor penetration depth and significant tissue autofluorescence thanks to the fluorescence in the visible-light spectrum (42).

### OTL38

OTL38, another folate analog, owes the major advantages of NIR dyes, like good penetration depth into solid tissue and slight autofluorescence in the NIR spectrum due to the decreased light dispersion and absorption in the blood. This promotes the differentiation between dye-rich tumor tissues and normal tissues that do not accumulate this folate analog, thereby the signal-to-noise ratio is significantly increased (45, 46).

### Other fluorophores

Other experimental fluorescence dyes, such as C700-OMe, have been found effective in NIR fluorescence imaging of costal cartilage in certain mice and pigs (47). Park et al. produced a series of oxazine derivates and found that oxazine 4 (Ox4) likely binds to the myelin sheath (48). Unfortunately, these dyes are still in the experimental phase, and no fluorophores have been successfully developed to specifically target the thymus.

## NIR fluorescence-guided surgery of the lung

Identifying small nodules and performing adequate lymphadenectomy while performing parenchyma-preserving radical surgery without complications are challenging for thoracic surgeons (48–50).

### Segmentectomy-intersegmental plane identification

Traditionally, the inflation and deflation technique has been used for intersegmental plane identification, owing to difficulties in emphysematous lungs and obstruction of the surgical view mainly during VATS procedures. To avoid these problems, other techniques have been developed, such as selective bronchoscopic ventilation of the affected bronchus, inflation of the selected bronchus by instilling oxygen through a butterfly needle, slip-knot ligation of the bronchus, or selective dye administration into the segmental pulmonary bronchus or artery (51–55).

**Figure 1:**
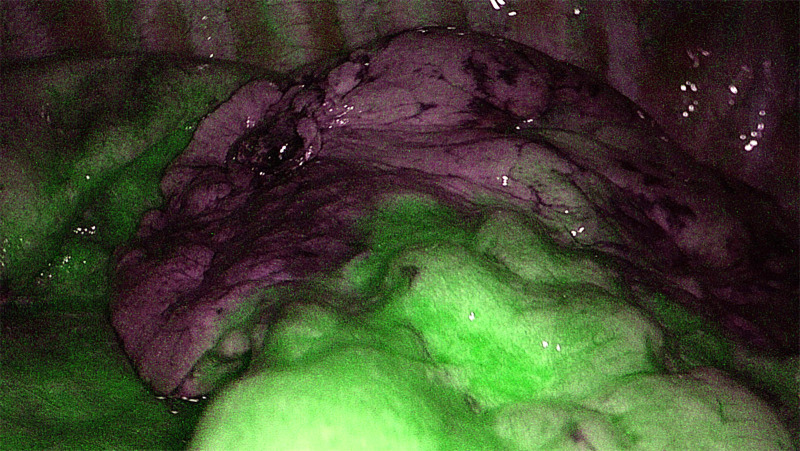
Intersegmental plane identification using ICG during VATS segmentectomy.

In 2009, Misaki et al. conducted an experimental study on dogs and demonstrated the feasibility of intersegmental plain identification using near-infrared imaging after intravenous administration of ICG (56). During the surgery, immediately after the identification and division of the segmental arteries, ICG was injected through a peripheral vein (56–63). The practical details are listed in Table 1. In emphysematous and bullous lungs, the blood flow is lower than that in the normal lung; therefore, visualization is more problematic, requiring a repeat and a higher dose of ICG (64). All authors emphasized the importance of proper preoperative evaluation with multiplanar CT and 3D reconstruction to identify segmental arterial branches because anatomical variations are quite frequent. Bedat et al. reported that NIR angiography results modified the surgical technique in 10% of patients. Additional arterial branch ligation or more extensive parenchymal resection has been indicated (61). In conclusion, NIR angiography is a safe, easy-to-reproduce, effective, and inexpensive method to improve the quality of VATS segmentectomy (Figure 1). Altough there is an animal study of intersegmental plane identification by direct injection of the dye into the segmental pulmonary arteries (54), there has been no study about using the fluorescence dye by this technique among clinical circumstances, and all authors have used the previously summarized the negative staining method.

**Table 1:** Different applications of near-infrared fluorescence-guidance in thoracic surgery.

LUNG						
	Study	Year	Dose	Administration route	Interval	Number of patients
Intersegmental plane	Misaki (56)	2009	25 mg	iv.	30–90s	dogs
	Mun (57)	2017	0,25 mg/kg		30–90s	22
	Guigard (58)	2017	25 mg			24
	Meacci (59)	2018	12,5–17,5 mg			
	Pschlik (60)	2018	0,15 mg/kg			86
	Bedat (61)	2018	12,5 mg			67
	Chen (62)	2019	25 mg			19
	Jin (63)	2019	0,5 mg/kg			21
	Motono (64)	2019	5 mg			22
	Yotsukura (65)	2021	0,25 mg/kg			209
	Sun (66)	2021	5 mg			198
	Oh (67)	2013	25 mg	Intrabronchial		40
	Wada (68)	2020	10–15 mg	Intrabronchial		15
	Sekine (55)	2012	10 mg	iv.		10
Pulmonary nodule identification	Doo (69)	2015	0,2 ml	Intratumoral inj.		34
	Ujie (70)	2017	0,15 ml	Intratumoral inj.		20
	Wen (71)	2018	0,5 ml	Intratumoral inj.		26
	Wu (72)	2021	1–2,5 mg	Intratumoral inj.		32
	Jiang (73)	2015	0,7–10 mg/kg	iv.	1 min-72 h	mice
	Okusanya (74)	2014	5 mg/kg	iv.	24 h	16
	Predina (78)	2017	OTL38:0,025 mg/kg iv.		3–6 h	20
	Kim (79)	2016	1 mg/kg		24 h	11
	Hamaji (80)	2019	0,25 mg/kg	iv.	12–24 h	22
	Predina (81)	2019	5 mg/kg	iv.	24 h	30
Sentinel lymph node	Yamashita (84)	2011	10 mg	Peritumoral	10 min	31
	Gilmore (87)	2021		Peritumoral	before surgery	29
	Hachey (88)	2017	0,5 ml	Peritumoral (bronchoscopy)	before surgery	20
	Digesu (90)	2018	0,5 ml	Peritumoral		42
Thoracic duct	Kamiya (91)	2009	7,5 mg	Bilateral inguinal	14 min	1 (case report)
	Matsutani (92)	2014	7,5 mg	Bilateral inguinal	10 min	1 (case report)
	Vecchiato (93)	2020	1,5 mg/kg	Bilateral inguinal lymph nodes	10,5 min	19

ICG can also be injected into the segmental bronchi. Oh et al. reported about a segmentectomy technique (67). In their prospective study during video-assisted open lung segmentectomy, after ligation or stapling of the segmental artery, vein, and bronchus, they injected ICG into the peripheral bronchus; thus, they were able to visualize the intersegmental border not only at the lung surface but also in the deep lung parenchyma. This technique has not become popular, perhaps because it first requires the division of all hilar structures. Wada et al. used an ultrathin bronchoscope to inject ICG into the targeted segmental or subsegmental bronchus immediately after intubation (68).

### Pulmonary nodule identification

In clinical practice, the intraoperative identification of small nodules during VATS is often difficult because of the loss of tactile feedback. Different methods have been developed to localize these lesions, such as preoperative microcoils, hookwire implantation, and dye injection (55, 69). Several authors have reported the use of ICG injection into the tumor preoperatively, with promising results (70–72). Uije et al. injected ICG and deployed a coil into the tumor, and after fluoroscopic localization, VATS resection was performed as a standard procedure (70). The NIR signal was detected in 90% of cases, and this method was found to be problematic when the tumor was deeply located (4.8 cm of the pleura) or when they had difficulties in deflating the lung.

Jiang et al. showed that ICG can be used for NIR imaging of lung tumors owing to the EPR effect (73). Okusenya et al. also used this method and performed open thoracotomy (74). They identified all 18 primary pulmonary nodules and no additional lesions by palpation and inspection. Intraoperative NIR imaging was able to discover 16 of the 18 primary nodules and discovered five additional nodules (74). NIR imaging could detect nodules as small as 0.2-cm and a wide histological range of primary tumors, independent of metabolic activity, tumor grade, and vascularity. The only important factor was the depth of tumor localization; subcentimeter nodules were detectable when they were not deeper than 1 cm in the collapsed lung, and larger nodules could have been imaged slightly deeper in the lung. The same group investigated the use of EC17, a folate analog, as folate receptor alpha is highly expressed in lung adenocarcinoma cells (75). In their study, they were able to detect 92% of proven lung adenocarcinomas using EC17 after resection, and the undetected tumors did not express folate receptors (76). Using OTL38, they reported improved specificity compared with that of ICG, improved depth of penetration compared with that of EC17, and presence of small, malignant additional nodules not seen on preoperative imaging (78). In a pilot study, Kim et al. used a lower dose (1 mg/kg) of ICG administration 24 h before surgery and after resection, and all specimens were examined for fluorescence signalling (77–80). In most patients, fluorescence signals could have been detected even in tumors as small as 3 mm. Fluorescence intensity was independent of the size, depth, metabolic activity, and pathology of the tumor; however, because of the passive accumulation of ICG, it was not possible to distinguish between a tumor and a inflammatory lesion.

By administering high doses (5 mg/kg of ICG) intravenouslythe day before surgery, Predina et al. could detect 89.1% of pulmonary sarcoma metastases and NIR imaging aided in detecting 24 additional and otherwise undetectable nodules in 20 patients (81). Nodule fluorescence did not depend on the histologal type but was best suited for tumorss at a maximum distance of 2 cm from the pleura.

### Sentinel lymph node identification

The concept of sentinel lymph node mapping has been successfully incorporated into the treatment of many solid tumors; but unfortunately, there is no fully reliable method available for sentinel lymph node evaluation in non-small cell lung cancer. An estimated 20% of sentinel lymph nodes bypass the closest lymph node station and skip to the mediastinal nodes. By failing to sample the hilar and mediastinal nodes, we can easily miss these skip metastases, and untreated occult micrometastatic disease results decrease in survival and an increase in recurrent tumor (82, 83). The use of blue dye and isotopes was not successful because of the anthracotic nodes and procedural feasibility.

Yamashita et al. were the first to report a series of NIR fluorescence-guided lung cancer sentinel lymph node mapping using ICG (84). ICG (10 mg) was administered peritumorally and sentinel lymph nodes were detectable in 80.3% of the patients; ICG leakage was the main reason for the failure. In an animal study, Soltesz et al. examined the feasibility of intraparenchymally injected NIR quantum dots (85, 86) and successfully identified sentinel lymph nodes in 100% of injections.

ICG is the most extensively investigated fluorophore for sentinel lymph node mapping. Gilmore et al. investigated the safety and feasibility of NIR imaging by administering peritumoral and subpleural injections of ICG in 29 patients who underwent thoracotomy and VATS lung resection (87). After the ICG injection, a short interval of lung ventilation was necessary to improve lymphatic drainage and at least 5 min for up to 20 min of lymphatic mapping was satisfactory in most cases. In a prospective study published by the same group, to avoid ICG spillage and other technical problems, 20 patients underwent navigational bronchoscopy-guided marking with ICG of lung lesions and achieved an 80% sentinel lymph node detection rate (88). The sentinel lymph node pathologic status was 100% sensitive and specific for overall nodal status. Hachey et al. also proved that NIR-guided sentinel lymph node status is 100% concordant with the final overall pathological nodal status, and extensive analysis of the sentinel lymph node can improve the detectabilty of micrometastasis (88–90). Thus NIR fluorescence guided sentinel lymph node mapping leads to upstaging of the tumor and initiate adjuvant therapy (88–90).

### Thoracic duct and chyle fistula identification

Thoracic duct and chyle fistula identification is often problematic because of inflammation and edema of the operative field after thoracic surgery. Several case reports (Ashite, Matsumati) have shown that inguinally administered ICG-guided fluorescence lymphography aided in successful visualization of the thoracic duct; thus, chyle fistula closure and thoracic duct ligation could have been performed by VATS, open, or even through the transabdominal approach (91–93). Vecchiato et al. reported that the identification rate of the thoracic duct was 100%, with a clear visualization of the duct, tributaries, and an aberrant duct (93). Fluorescence could have been detected until the end of the operation in all patients. They used ultrasound guidance for inguinal lymph node injection of 0.5 mg/kg if diluted ICG was used. They also stated that fluorescence guidance allows safe dissection and could help surgeons preserve the thoracic duct integrity.

## Conclusion and future perspectives

NIR fluorescence-guided imaging is an emerging new technique that provides various new methods in several fields of thoracic surgery. ICG is the most extensively used fluorophore and has the potential to improve patient management. Almost immediately after intravenous injection, it can identify intersegmental planes of the lung, making segmentectomies more accurate. After peritumoral injection, it can help identify sentinel lymph nodes and visualize the lymphatic drainage route during lung resections, thus providing proper lymphadenectomy and N staging or can help to treat complications such as chylorrhea by identifying the thoracic duct. NIR fluorescence-guided sentinel lymph node mapping can identify the sentinel lymph node correctly, facilitating the identification of micrometastases, thus providing correct staging and potentially better survival. Using the so-called EPR, a passive targeting effect, 24 h after high-dose administration of ICG, can visualize even small pulmonary nodules, filling the vacuum after the loss of tactile feedback during VATS. Other fluorescent dyes, such as OTL38, are more specific to adenocarcinomas but are not widely used.

In the future, it is expected that NIR will be more widely accepted and used worldwide and will improve patient care. Fluorescence dye development is an active area of research, and active targeting molecules and dyes will be developed that are specific to tumor cells; thus, NIR imaging will be more accurate. NIR fluorescence guided surgery is safety and easy, thus learning curve of this new method is smooth and quick and can be inserted into educational programes easily, which will help its worldwide spreading.

## Data Availability

The datasets [GENERATED/ANALYZED] for this study can be found in the [NAME OF REPOSITORY] [LINK]. Please see the Data Availability section of the Author guidelines for more details.

## Additional requirements

For additional requirements for specific article types and further information please refer to Author Guidelines.
