# Supplementary material for: Near-infrared fluorescence guided surgery: State of the evidence from a health technology assessment perspective
Source: Front Surg. 2022 Jul 26;9:919739. doi: 10.3389/fsurg.2022.919739 (PMC9360526; doi:10.3389/fsurg.2022.919739)
Supplement: Supplementary file 1 [file Table_1_v1.docx]

| **LUNG** | | | | | | |
| --- | --- | --- | --- | --- | --- | --- |
|  | **Study** | **Year** | **Dose** | **Administration route** | **Interval** | **Number of patients** |
| **Intersegmental plane** | Misaki (56) | 2009 | 25mg | iv. | 30-90s | dogs |
|  | Mun (57) | 2017 | 0,25mg/kg | | 30-90s | 22 |
|  | Guigard (58) | 2017 | 25mg |  |  | 24 |
|  | Meacci (59) | 2018 | 12,5-17,5mg | |  |  |
|  | Pschlik (60) | 2018 | 0,15mg/kg | |  | 86 |
|  | Bedat (61) | 2018 | 12,5mg |  |  | 67 |
|  | Chen (62) | 2019 | 25mg |  |  | 19 |
|  | Jin (63) | 2019 | 0,5mg/kg |  |  | 21 |
|  | Motono (64) | 2019 | 5mg |  |  | 22 |
|  | Yotsukura (65) | 2021 | 0,25mg/kg | |  | 209 |
|  | Sun (66) | 2021 | 5mg |  |  | 198 |
|  | Oh (67) | 2013 | 25mg | intrabronchial |  | 40 |
|  | Wada (68) | 2020 | 10-15mg | intrabronchial |  | 15 |
|  | Sekine (69) | 2012 | 10mg | iv. |  | 10 |
|  |  |  |  |  |  |  |
| **Pulmonary nodule identification** | Doo (71) | 2015 | 0,2ml | intratumoral inj. |  | 34 |
|  | Ujie (72) | 2017 | 0,15ml | intratumoral inj. |  | 20 |
|  | Wen (73) | 2018 | 0,5ml | intratumoral inj. |  | 26 |
|  | Wu (74) | 2021 | 1-2,5mg | intratumoral inj. |  | 32 |
|  | Jiang (75) | 2015 | 0,7-10mg/kg | iv. | 1min-72h | mice |
|  | Okusanya (76) | 2014 | 5mg/kg | iv. | 24h | 16 |
|  | Predina (80) | 2017 | OTL38:0,025mg/kg iv. | | 3-6h | 20 |
|  | Kim (81) | 2016 | 1mg/kg |  | 24h | 11 |
|  | Hamaji (82) | 2019 | 0,25mg/kg | iv. | 12-24h | 22 |
|  | Predina (83) | 2019 | 5mg/kg | iv. | 24h | 30 |
|  |  |  |  |  |  |  |
| **Sentinel lymph node** | Yamashita (86) | 2011 | 10mg | peritumoral | 10min | 31 |
|  | Gilmore (89) | 2021 |  | peritumoral | before surgery | 29 |
|  | Hachey (90) | 2017 | 0,5ml | peritumoral (bronchoscopy) | before surgery | 20 |
|  | Digesu (92) | 2018 | 0,5ml | peritumoral |  | 42 |
|  |  |  |  |  |  |  |
|  |  |  |  |  |  |  |
| **Thoracic duct** | Kamiya (93) | 2009 | 7,5mg | bilateral inguinal | 14min | 1 (case report) |
|  | Matsutani (94) | 2014 | 7,5mg | bilateral inguinal | 10min | 1 (case report) |
|  | Vecchiato (95) | 2020 | 1,5mg/kg | bilateral inguinal lymph nodes | 10,5min | 19 |
|  |  |  |  |  |  |  |
